# Network Pharmacology Approaches Used to Identify Therapeutic Molecules for Chronic Venous Disease Based on Potential miRNA Biomarkers

**DOI:** 10.3390/jox14040083

**Published:** 2024-10-15

**Authors:** Oscar Salvador Barrera-Vázquez, Juan Luis Escobar-Ramírez, Gil Alfonso Magos-Guerrero

**Affiliations:** Department of Pharmacology, Faculty of Medicine, University National Autonomous of Mexico (UNAM), Mexico City 04510, Mexico; osbarrerav@comunidad.unam.mx (O.S.B.-V.); jlescobar@comunidad.unam.mx (J.L.E.-R.)

**Keywords:** chronic venous disease, miRNAs, network, biomarkers, genes, molecules, contaminants

## Abstract

Chronic venous disease (CVD) is a prevalent condition in adults, significantly affecting the global elderly population, with a higher incidence in women than in men. The modulation of gene expression through microRNA (miRNA) partly regulated the development of cardiovascular disease (CVD). Previous research identified a functional analysis of seven genes (CDS2, HDAC5, PPP6R2, PRRC2B, TBC1D22A, WNK1, and PABPC3) as targets of miRNAs related to CVD. In this context, miRNAs emerge as essential candidates for CVD diagnosis, representing novel molecular and biological knowledge. This work aims to identify, by network analysis, the miRNAs involved in CVD as potential biomarkers, either by interacting with small molecules such as toxins and pollutants or by searching for new drugs. Our study shows an updated landscape of the signaling pathways involving miRNAs in CVD pathology. This latest research includes data found through experimental tests and uses predictions to propose both miRNAs and genes as potential biomarkers to develop diagnostic and therapeutic methods for the early detection of CVD in the clinical setting. In addition, our pharmacological network analysis has, for the first time, shown how to use these potential biomarkers to find small molecules that may regulate them. Between the small molecules in this research, toxins, pollutants, and drugs showed outstanding interactions with these miRNAs. One of them, hesperidin, a widely prescribed drug for treating CVD and modulating the gene expression associated with CVD, was used as a reference for searching for new molecules that may interact with miRNAs involved in CVD. Among the drugs that exhibit the same miRNA expression profile as hesperidin, potential candidates include desoximetasone, curcumin, flurandrenolide, trifluridine, fludrocortisone, diflorasone, gemcitabine, floxuridine, and reversine. Further investigation of these drugs is essential to improve the treatment of cardiovascular disease. Additionally, supporting the clinical use of miRNAs as biomarkers for diagnosing and predicting CVD is crucial.

## 1. Introduction

Chronic venous disease (CVD) is a widespread disease estimated to affect 83.6% of elderly individuals globally. This disease, which includes varicose veins and chronic venous insufficiency (CVI), is a prevalent medical condition among adults. CVD affects individuals worldwide, with the highest incidence in developed countries [1]. Reports describe venous leg ulcers as the most severe stage of chronic venous disease (CVD) [2]. CVD has significant consequences for individuals and society, affecting the quality of life through the experience of pain, heaviness, itching, and reduced mobility [3]. The most common cause of CVI is varicose veins in the lower extremities, with the most severe form resulting in venous ulceration. It is estimated that 30–40% of the adult population has varicose veins, and up to 6% of patients with varicose veins develop ulcers. In addition, 30% of varicose veins can develop into more severe forms of CVI.

The pathophysiology of CVD involves varicose veins, dysfunction of venous valves, and skin ulceration resulting from impaired cell proliferation, apoptosis, and angiogenesis [4]. Vascular cell functions, including cell differentiation, proliferation, migration, and apoptosis, are decisively regulated by microRNA (miRNA)-dependent gene expression modulation [5,6]. Many studies have revealed that miRNAs’ aberrant expression is associated with pathological conditions’ development [7]. In vascular disease, miRNAs have demonstrated modulatory functions in angiogenesis, endothelial cell dysfunction, and response to ischemic events [8]. Several miRNAs are regarded as potential biomarkers for cardiovascular diseases [8]. miRNAs and genes exhibit distinctive expression patterns in venous samples from patients with CVI compared to healthy patients [9,10,11,12]. Some works have separately proposed some biomarkers that could be used within CVD, and through functional analysis, seven genes (CDS2, HDAC5, PPP6R2, PRRC2B, TBC1D22A, WNK1, and PABPC3) selected as miRNA targets by in silico methods were recognized as potential biomarkers of CVD. However, their complete understanding remains elusive [13].

microRNAs (miRNAs) are small non-coding RNAs, typically 21–25 nucleotides in length, originating from both coding and non-coding transcription units found in genic (intronic or exonic) and intergenic regions [14,15]. They are pivotal in regulating essential processes such as tissue homeostasis and cell signaling, operating at the post-transcriptional gene expression level [16]. These molecules also have an extracellular role and can be released into microvesicles, modulating gene expression in several tissues [17,18,19]. Both intracellular and extracellular miRNAs can be detected in tissue samples and various biological fluids, including serum, plasma, urine, saliva, sweat, and tears. However, this approach remains unexplored, and is mainly used in personalized medicine as a diagnostic or therapeutic tool.

Network representations have proven essential in depicting interactions among entities of interest across various domains. This tool is precious for analyzing and visualizing complex biological processes [20]. Leveraging known interactions discovered from previous experiments creates biological networks. These intricate molecular interaction networks provide valuable insights into biological processes, enabling us to pinpoint critical nodes and other topological features associated with complex phenotypes in health and disease [21]. According to network medicine theory, disease-associated phenotypes are hypothesized to result from perturbations in gene networks rather than individual gene mutations acting in isolation [22].

Restoring or inhibiting specific miRNAs in diseased cells can be accomplished through various strategies, including (1) delivering miRNA mimics or inhibitors using modified nucleic acids, (2) modifying miRNAs using viral delivery vector systems, (3) delivering miRNA mimics or inhibitors using nonviral delivery vector systems, and (4) developing small-molecule drugs that target microRNAs [23]. A further strategy has been using drugs, natural products, or plant extracts, which have been shown to modulate the expression of miRNAs through multiple mechanisms in various pathologies [24,25,26,27].

Hesperidin, a drug widely used in the treatment of CVD [28,29,30], has been demonstrated to enhance endothelial function by increasing the availability of nitric oxide (NO) while simultaneously suppressing the production of reactive oxygen species (ROS) and plasma levels of pro-inflammatory markers [31]. This flavonoid has been involved in modulating miRNAs by downregulating miR-21-5p and upregulating miR-16-5p and miR-34a-5p [32], which have been involved in the physiopathology of several cardiovascular diseases, including CVD [4,33,34,35], and has been considered to be used as a reference compound in our study.

In this sense, because of the critical participation of miRNA in gene expression in the development of CVD, we focused on identifying potential miRNAs and drugs that could modulate the expression of those miRNAs and may antagonize their profile expression during CVD. To achieve this aim, we employed a network-based approach. The data were collected from both the literature and miRNA-related databases to identify, by network analysis, potential molecules that interact with miRNAs regulated by hesperidin and that modify the gene expression involved in CVD derived from miRNAs collected from several studies with potential miRNA biomarker candidates and as potential therapeutic agents [4,9,11,36,37,38,39,40].

## 2. Materials and Methods

### 2.1. Reviewing

#### Bibliographic Screening to Select MicroRNAs Involved in CVD

We searched the PubMed database from 2016 to 2024 using MESH terms such as microRNAs, miRNA, chronic venous disease, and chronic venous insufficiency. Two independent reviewers selected studies that met the eligibility criteria.

The bibliography considered in this work includes studies about miRNAs with significant expression profiles in CVD reported between 2016 and 2024, in vitro studies that included cases and control groups, and tests in human samples with validated CVD diagnoses. Studies not performed in English that do not include controls or duplicated data, studies conducted in animal models, or studies conducted in human-derived sources were excluded from this research.

### 2.2. Data Collection

We sought to analyze the genes influenced by the miRNAs identified through data extraction in both the reported bibliography (validated) and servers (predicted) from the Target Expression Analysis section of the miRDB-MicroRNA Target Prediction Database (http://www.mirdb.org/mirdb/index.html, accessed on 1 May 2024), considering those targets with a prediction score > 95%. The network analysis used the mirNET database to search for small molecules responsible for modulating CVD-associated miRNAs (https://www.mirnet.ca, accessed on 13 May 2024). The bar plot was generated using the ggplot2 package in R, leveraging information sourced from the literature and the Target Expression Analysis section of the miRDB-MicroRNA Target Prediction Database (http://www.mirdb.org/mirdb/index.html, accessed on 28 June 2024).

### 2.3. Network Structural Analysis

We employed Cytoscape software (v.3.10.2) to construct networks aiming to identify the most connected miRNAs and relevant pathways within the context of CVD [41]. The Cytohubba plugin was used to identify the most interconnected genes within the network [42]. To elucidate the potential principal signaling pathways modified by the miRNAs, we employed the JEPETTO plugin within Cytoscape [43]. The pathways exhibiting the most significant enrichment (*p* < 0.05) were adjusted using their XD scores based on the insights from the previous report [44].

### 2.4. Network Pharmacology Analysis

We utilized the Cytoscape software (v.3.10.2) to construct the structural networks [41] to analyze the information from the mirNET database and identify the chemical compounds responsible for modulating miRNAs involved in CVD (https://www.mirnet.ca, accessed on 29 June 2024). The Cytohubba plugin was used to identify the most connected genes in the network. [42]. Furthermore, the highly interconnected small molecules were identified based on their scores, and an in-depth exploration of their respective functions was conducted in the literature.

To search for molecules with the same targeted miRNAs as hesperidin, (1) we generated a dataset of miRNAs targeted by hesperidin. These miRNAs were obtained from the SM2miR server (http://www.jianglab.cn/SM2miR/, accessed on 30 June 2024). This server allows us to identify whether those miRNAs targeted by hesperidin were up- or downregulated. (2) Subsequently, we obtained each sequence of those miRNAs upregulated or downregulated by hesperidin, searching the miRNA accession number in the miRbase server (https://www.mirbase.org/, accessed on 1 July 2024) [45]. (3) We analyzed each miRNA sequence and predicted other molecules that also regulate those same miRNAs in the PSRR (Prediction of SM-miRNA Regulation pairs by random forest) server (https://rnadrug.shinyapps.io/PSRR/, accessed on 2 July 2024). Using this random forest algorithm, we achieved the highest AUC values of 0.911 for the upregulation model and 0.896 for the downregulation model on the testing datasets, outperforming four other machine learning algorithms [46]. These molecules were employed to construct a structural network using the Cytoscape software (v.3.10.2) [41], and the results were analyzed with the Cytohubba plugin [42]. We considered those molecules with high degree centrality, which tends to highlight nodes (molecules) with higher degrees (more direct connections), and betweenness centrality, to identify those nodes (molecules) that lie on critical paths between others.

### 2.5. Using QED Index to Determine Drug-like Candidates

To determine the drug-likeness using the quantitative estimate of drug-likeness (QED), we used the DruLito software (https://drulito.software.informer.com/, accessed on 4 July 2024), as previously described [47]. This index has enabled us to identify compounds with favorable oral administration characteristics [48]. The DruLito software (version 1.0.0) evaluated SDF files from PubChem. Our analysis focused solely on molecules that were accessible via this server. The analytical data are reported in Supplementary Materials Tables S1–S10.

## 3. Results

**Scheme 1:**
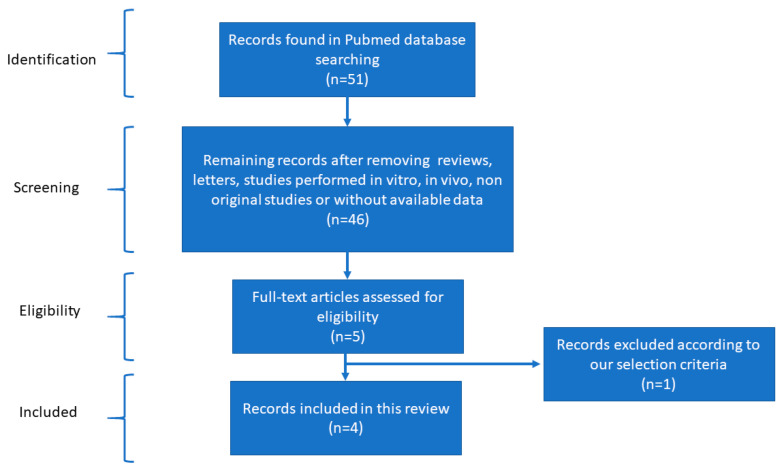
Flow diagram of the bibliographical screening performed for this research.

**Figure 1:**
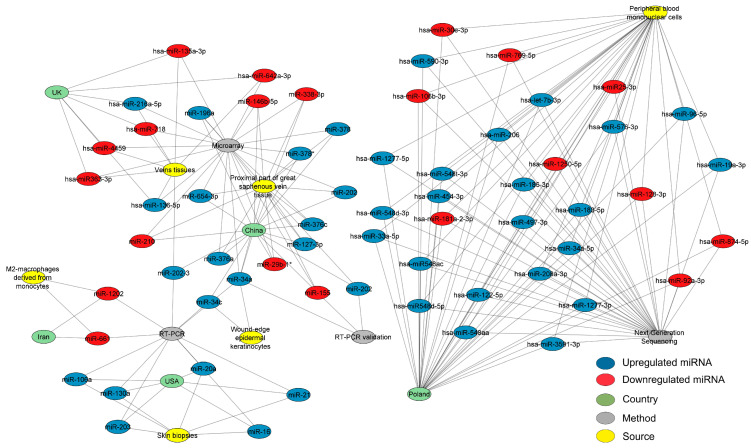
Network analysis of CVD-associated miRNAs with their expression, sources, countries of origin, and detection methods. The network depicts the interconnected structure of miRNAs derived from CVD patients, organized by their expression, sources, countries of origin, and detection methods. In the network, miRNAs are denoted as blue (upregulated) or red (downregulated) nodes, green nodes represent countries, yellow nodes represent sources, and gray nodes represent detection methods. The connections between the nodes signify the frequency of independent study reports. The three most outstanding sources were the proximal part of the significant saphenous vein tissue, vein tissues, and peripheral blood mononuclear cells. China was the country with the most available finds from miRNAs. Microarrays and RT-PCR are the most effective methods for diagnosing CVD. At least two tissues are expected to contain five specific miRNAs: miR-34a, miR-34c, miR-202-3, miR-1202, and miR-130a. The network, constructed using Cytoscape software (v.3.10.2), comprises 78 nodes and 193 edges, with a diameter and a network density of 6 and 0.106, respectively. Please refer to the Supplementary Materials (Figure S1) for a better image resolution.

After reviewing the literature and applying the filters mentioned in the methods for bibliographical screening, we created different networks using the selected studies. Scheme 1, according to the PRISMA statement, outlines the article selection criteria for subsequent analysis. A carefully selected database of differentially expressed miRNAs in cardiovascular disease was acquired to determine the most pertinent miRNAs. A structural network analysis was built to find the common miRNAs between the countries where these studies were performed. The data for this network analysis were collected from studies where the detection methods, tissues, and expression of each miRNA were found (Figure 1). The three most outstanding sources were the proximal part of the significant saphenous vein tissue, vein tissues, and peripheral blood mononuclear cells (PBMCs). This network analysis found that Poland researched CVD-associated miRNAs most, followed by China, the UK, and the USA. The most effective methods for diagnosing CVD were microarray and RT-PCR. The analysis identified several interconnected miRNAs within the network, including miR-34a, miR-34c, miR-202-3, miR-1202, and miR-130a. The analysis detected miR-34a in wound-edge epidermal keratinocytes and the proximal part of the significant saphenous vein tissue using microarray, RT-PCR, and RT-PCR validation methods.

**Figure 2:**
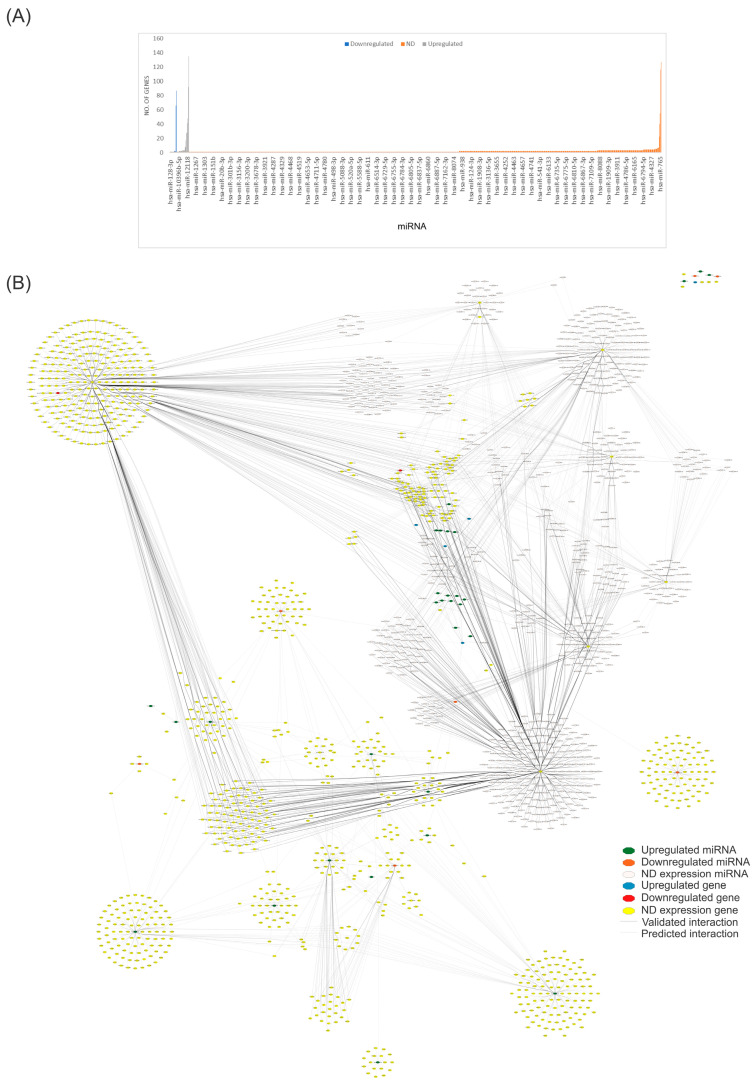
Network analysis of miRNAs associated with CVD and their predicted targets. (**A**) The bar plot visually presents the number of targetable genes in the miRNA curated dataset. In the plot, gray bars represent upregulated genes, blue bars denote downregulated genes, and orange bars indicate genes with undetermined expression (ND). (**B**) We constructed a structural network using reported and predicted interactions between miRNAs and their targeted genes. This network consists of 1882 nodes and 5267 edges, with a diameter and a network density of 12 and 0.001. The network was created using Cytoscape software (v.3.10.2). Please refer to the Supplementary Materials (Figure S2) for a better image resolution.

To identify the miRNAs that regulate the most significant number of genes and to find critical miRNAs that could regulate numerous genes, we utilized our complete dataset of miRNAs (Table S1) from the miRBD database (http://www.mirdb.org/mirdb/index.html, accessed on 28 June 2024) to identify genes and pathways that interact with them. We only considered target genes with a score above 95% (Figure 2). Figure 2A depicts the genes regulated by each miRNA involved in CVD found in the miRBD database, including miR-593-3p (135 genes), miR-19a-3p (127 genes), miR-92a-3p (116 genes), miR-301a-3p (92 genes), and mir-181a-2-3p (87 genes). Figure 2B depicts the number of nodes (genes) interacting with the whole miRNA dataset and shows the most regulated targets indicated by the miRBD database. Using this new set, we created another network with the genes altered by these miRNAs.

**Figure 3:**
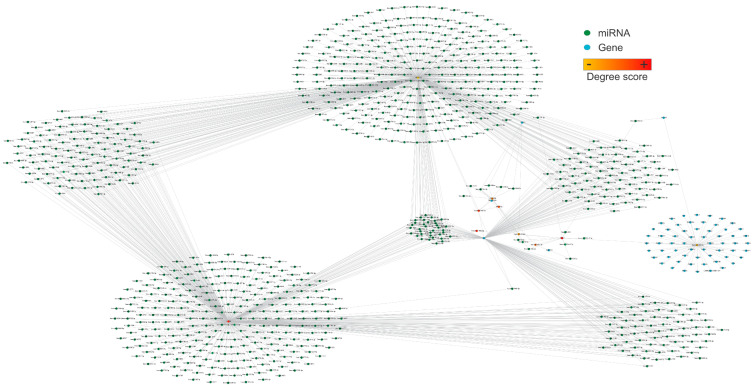
The structural network represents the ten most connected nodes, including miRNAs and targets. Nodes are color-coded from orange to yellow based on their degree of connection, representing the most connected genes and miRNAs in the network. The most relevant nodes in this network are WNK-1, hsa-miR-106b-3p, IL1NR, hsa-miR-92a-3p, PPP6R2, hsa-miR-454-3p, PRRC2B, hsa-miR-548ac, hsa-miR-128-3p, and ADIPOQ. The network consists of 921 nodes and 1256 edges, with a diameter and a network density of 7 and 0.003, respectively. This network was created using Cytoscape software (v.3.10.2). Please refer to the Supplementary Materials (Figure S3) for a better image resolution.

We further analyze the structural network in Figure 2, which shows the most connected nodes. The most connected genes were WNK-1, IL1RN, PPP6R2, PRRC2B, and ADIPOQ, and the most connected miRNAs were hsa-miR-106b-3p, has-miR-92a-3p, hsa-miR-454-3p, hsa-miR-548ac, and hsa-miR-128-3p (Figure 3).

**Figure 4:**
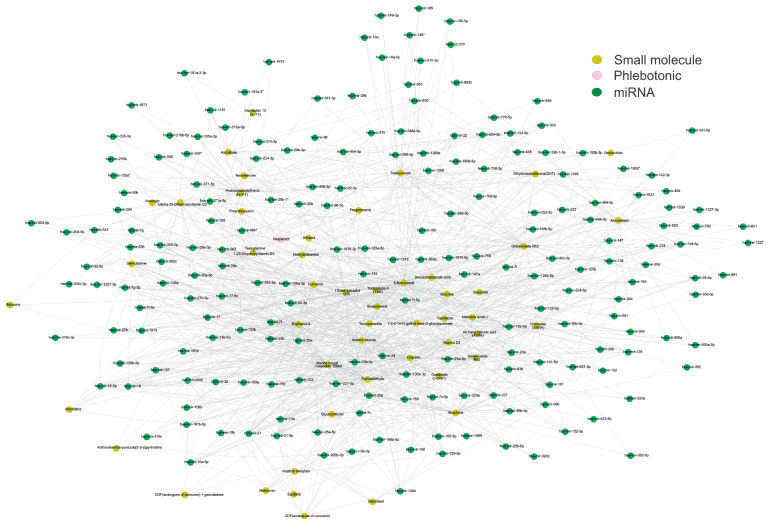
The structural network of small molecules, phlebotonic, and miRNAs. The structural network depicts the targeted miRNAs (green nodes) between small molecules (yellow nodes) and the phlebotonic hesperidin (pink node). It was observed that curcumin exhibited the highest connection of miRNAs with the reported phlebotonic hesperidin. The network involves 246 nodes and 1153 edges, with a diameter and a network density of 6 and 0.038, respectively. The network construction utilized Cytoscape software (v.3.10.2). Please refer to the Supplementary Materials (Figure S4) for a better image resolution.

After defining these most connected and relevant genes involved during CVD, they were used to perform an enrichment analysis to better understand the pathophysiology of CVD. The targeted genes of the miRNAs involved in CVD were analyzed to correlate CVD with other pathways or processes. Table 1 lists the most relevant pathways or processes associated with CVI. Lately, a pharmacology network analysis was conducted considering the most relevant miRNAs from the dataset and the compounds present in the mirNET database (https://www.mirnet.ca/upload/MirUploadView.xhtml, accessed on 29 June 2024) (Figure 4 and Table 2). This analysis was performed to find small molecules of different natures that could interact with our most relevant miRNAs. Interestingly, the interactions inside the network showed that drugs and toxicants could modulate such miRNAs. This network showed that the reported phlebotonic drug hesperidin shares several miRNAs with the natural compound curcumin. Table 2 displays the most relevant features of each compound. Furthermore, the interactions within the network suggest that drugs and toxicants have the potential to modulate these miRNAs. As a result, it is crucial to consider their influence on toxicological analysis and in conducting further ecological and epidemiological studies (Table 3).

**Figure 5:**
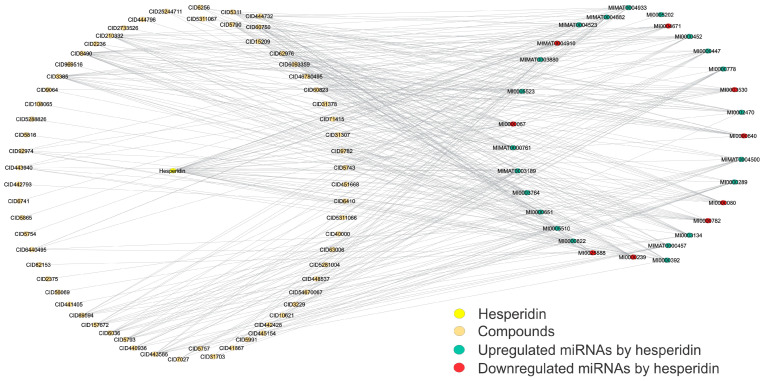
Structural network of hesperidin-specific miRNAs that are also altered by small molecules. This structural network depicts the miRNA profiles shared by both miRNAs that are specifically upregulated or downregulated by the reference compound hesperidin and the small molecules studied in this work. These shared miRNA profiles between hesperidin and small molecules allow the selection of potential candidates for CVD treatment. Downregulated miRNAs are shown in red, upregulated in blue, and those shared with small molecules in orange. The network involves 93 nodes and 320 edges, with a diameter and a density of 4 and 0.075, respectively. This network was created using Cytoscape software (v.3.10.2). Please refer to the Supplementary Materials (Figure S5) for a better image resolution.

To further analyze the interactions between the miRNAs targeted by hesperidin, determining if this molecule influences their regulation, and thus determine new candidates that can also share and similarly regulate these miRNAs targeted by hesperidin, we generated a network structural analysis to determine those molecules that were most connected and shared more intermediates within this network (Figure 5). For more information on how this network was built, see the Materials and Methods. Table 4 shows the degree and betweenness values of the most relevant molecules in this network (20 molecules).

Finally, we determined their drug-likeness through the QED index to analyze whether these most relevant molecules could be suitable candidates for oral administration. At the end of the analysis of the eighteen molecules obtained from the study of the structural networks that share miRNAs with hesperidin, only six met this QED index criteria of a value greater than 0.5, identifying desoximetasone, curcumin, flurandrenolide, fludrocortisone, diflorasone, and reversine as the most suitable candidates to be administered orally in this study (Table 4).

## 4. Discussion

CVD is a multifactorial condition characterized by complex pathophysiological mechanisms [40], among which the non-coding RNAs, such as miRNAs, regulate multiple functions in biological and pathological processes, positively or negatively, promoting or not the action of the corresponding mRNA product. Numerous miRNAs can be biomarkers for several diseases and conditions, including cardiovascular diseases, cancer, and sepsis [92]. MicroRNAs (miRNAs) can be released into the bloodstream, where they exist as stable circulating free miRNAs or remain safeguarded from endogenous RNAse enzyme activity through encapsulation in exosomes or proteins [93]. Numerous reports have described the significant role of miRNAs in processes closely associated with vascular pathology, such as angiogenesis, endothelial dysfunction, and vascular inflammation. Therefore, dysregulation of the miRNA expression profiles could be considered a promising biomarker of CVD [94].

Although few studies have been carried out, most of these studies come from the USA, Poland, China, and the UK. However, there were no studies in Latin America, so many aspects of CVD and miRNAs are still unknown in this population. Thus, urgent research must be translated into clinical practice.

One of the most connected miRNAs is miR-34a. This miRNA has been extensively studied in cancer biology in in vitro conditions due to its role as a tumor suppressor. During cancer development, this miRNA has been demonstrated to impact several crucial processes, including cell cycle progression, self-renewal, senescence, apoptosis, epithelial-to-mesenchymal transition, differentiation, migration, and metastasis. Evidence indicates that miR-34a is a crucial regulator of age-related tissue modifications and a promoter of cellular senescence. Levels of miR-34a have shown an increase with age. This increase is found in several organs, including the muscle, heart, and aorta, and it promotes detrimental organ remodeling and functional decline. In the cardiovascular system, miR-34a promotes the senescence of endothelial cells (ECs) in the vascular wall and vascular smooth muscle cells (VSMCs), principally by direct negative regulation of its best-known target, the longevity-associated gene sirtuin 1 (SIRT1) [95]. This pathway promotes arterial inflammation and the development of age-related VD.

Furthermore, miR-34a expression could be influenced by other risk factors promoting vascular changes similar to aging [95]. These features make this miRNA a suitable target for treating VD. It has been described as a target of flavonoids inhibiting miR-34a synthesis and upregulation of SIRT1 expression, protecting endothelial cells from senescence [96]. This finding correlates with the literature because most drugs to treat VD come from a flavonoid nature [30].

Furthermore, miR-34a targets XIAP [97], which has been implicated in resistance to apoptosis, a phenomenon possibly related to the downregulation of apoptosis in the medial layer of varicose veins [98]. This resistance to apoptosis has also been found in SASP (senescence-associated secretory phenotype), where varicose veins lead to vascular endothelium dysfunction, causing a pro-inflammatory environment [99]. Expression of miR-34a has shown significant upregulation in both the plasma and atherosclerotic plaques of patients diagnosed with CAD (coronary artery disease). This finding suggests that overexpression of miR-34a in our study is common in both arterial and venous pathology. Also, this result agrees with the research performed by Zalewski et al. [4], where this miRNA was found to be upregulated in patients with CVD. By integrating both experimental and predictive data analysis in our review, mir-34a is an outstanding miRNA and may be used as a potential biomarker for the early detection of CVD.

miR-34c has been associated with several cellular processes in the cardiovascular system [100], playing a protective role in hypoxia by activating autophagy through repressing BCL2. Significant cellular stress is produced during hypoxia that originates diverse pathological consequences, such as cardiovascular diseases and cancers [101].

miR-202-3p is downregulated during CVD. This downregulation is involved in metalloproteinase-1 (MMP-1) overexpression. MMP1 promotes trans-endothelial migration by degrading inter-endothelial junctions and disrupting endothelial integrity [102]. Thus, this loss in miR-202-3p expression may increase the activity of the MMP-1, which plays a role in CDV pathogenesis [103]. miR-1202 plays regulatory roles in MMP9 function during CVD development. However, it is required to evaluate their potential diagnostic and therapeutic utility [37]. This finding agrees with the results obtained by Zalewski et al., where miR-1202 is downregulated in patients with varicose veins. Thus, our findings also propose this miRNA as a valuable potential biomarker [94]. miR-130a was the most interconnected miRNA in this network. miR-130a has been reported in skin biopsies from venous ulcers, compared to standard skin specimens [94]. An association with the overexpression of these miRNAs has been established because they may inhibit wound healing by targeting LepR [4,94].

Interestingly, the other most connected miRNAs, miR-106b-3p, miR-92a-3p, miR-454-3p, miR-548ac, and miR-128-3p, were obtained by next-generation sequencing (NGS) of PBMCs of patients with varicose veins [94]. These findings allow us to know the relevance of NGS and PBMCs in diagnosing VD.

According to structural network analysis, the most connected genes were WNK-1, IL1NR, PPP6R2, PRRC2B, and ADIPOQ WNK1, which have been associated with downregulation of miR-181a-2-3p and miR-106b-3p in previous reports [4]. WNK1 plays a role in endothelial cell proliferation, chemotaxis, and invasion [104]. In mouse model studies, the lack of the WNK1 gene leads to embryonic death due to angiogenic and cardiovascular defects [104]. The hypothesis postulates that the upregulation of WNK1 is associated with the downregulation of miR-181a-2-3p and miR-106b-3p, thereby leading to the induction of inflammation and aging in response to oxidative stress in CVD [4]. Interleukin-1 receptor antagonist (IL1RN) has a biological role in the pro-inflammatory cytokine interleukin-1. This downregulation may be implicated in the chronic inflammation of the CVD [105]. The PPP6R2 gene is upregulated during CVD. This gene encodes proteins that act as a significant T-loop phosphatase for Aurora A, a crucial mitotic kinase. Aurora A activation is very complex and occurs at specific subcellular locations due to signaling events, including phosphorylation and oxidation [106]. Aurora A controls key oncogenic pathways associated with drug resistance and poor patient outcomes [106]. According to the findings of this study, several miRNAs are present in both CVD and some types of cancer. PPP6R2 may be a potential early biomarker for CVD and cancer.

*PRRC2B* has been reported as possibly upregulated by miR-92a-3p [4]. This gene-encoding protein is involved in brain development. As noted by Zalewski et al., specific neurodevelopmental processes might be implicated in the pathogenesis of CVD, highlighting potential associations between vascular and neurodegenerative disorders [4]. However, the network analysis showed another potential biomarker in this study, the ADIPOQ gene. ADIPOQ gene polymorphisms are related to adiponectin levels, insulin resistance, and metabolic diseases such as diabetes mellitus type II (DMT2) [107]. This finding also connects with WNK, which is involved in DMT2 [108,109] and vascular disorders [110]. In this context, evidence has been proposed that the incidence of diabetes mellitus is much higher in patients with CVI. More intense therapy directed against CVI in diabetic patients could potentiate the prevention of complications such as leg edema, deep vein thrombosis, progression of CVI, and post-thrombotic syndrome and improve the quality of life [111].

For the first time, small molecules (toxins and drugs) that regulate the expression of several miRNAs are involved in a CVD study through structural network analysis. Certain identified compounds include standard air or water contaminants and drugs that possess the potential to regulate miRNAs associated with CVD and its progression. Formaldehyde (FA), a prevalent air pollutant, is identified among xenobiotics and is widely encountered in nature, industrial processes, and various consumer products [67,112]. Its long-term exposure may provoke skin disorders, cancer, and cardiovascular disease [113]. This small molecule alters vascular function and oxidative stress, influencing cardiovascular health [113]. Exposure to FA from various sources can lead to heart diseases, including arrhythmia, myocardial infarction (MI), heart failure (HF), and atherosclerosis (AS) [112]. Acute FA exposure has influenced adult female vascular function in the arms and legs, decreasing conduit vessel function without altering microvascular function [113]. Also, FA causes an increase in blood pressure by activating the ACE/AT1R axis [114], so its exposure may be involved in the development of hypertension, which has been proposed as a potential cause of the early development of CVD [114,115,116,117,118]. Additional research is imperative to ascertain the diverse effects of varying concentrations and durations of FA exposure in both genders, older adults, and individuals who are particularly susceptible to cardiovascular disease, including CVD. Ethanol has been shown to induce vascular toxicity, linking patients with disorders associated with excessive ethanol consumption and the development of CVD. This process has been described through the modulation of the AT1 receptor with vascular hyper-contractility, promoting mitochondrial dysfunction, mtROS production, and reduced bioavailability of NO and H_2_O_2_ [119].

17beta-estradiol (E2) and diethylstilbestrol are estrogens [68,73]. Estrogens are critical regulators of vascular homeostasis, primarily functioning through ERα and ERβ, which serve as ligand-gated transcription factors. Research findings have revealed a substantial upregulation in the expression of ERα, ERβ, and GPER in the tissue of individuals with CVD. This upregulation has been associated with disease severity and has shown a correlation with the clinical stage of VD [120]. In addition, a large group of heterogeneous drugs with anticancer properties showed significant interactions with the miRNAs proposed by our network analysis. These results could be because most of the miRNAs in this study are involved in the development of, or are potential targets of, some cancers [102,121,122,123,124,125,126,127,128,129]. Our data agree with the evidence that shows that CVD may have a relationship with the development of cancer [130,131]. These results encourage more extensive and in-depth studies to be carried out to elucidate the pathogenesis mechanisms shared by both CVD and the development of some types of cancer.

Among the compounds that share those miRNAs that are targeted by the reference compound, hesperidin, we find a group of heterogeneous molecules of different types, such as corticosteroids, diarylheptanoids, antivirals, nucleoside analogs, oncology drugs, purine derivatives, estrogen receptor modulators, histone deacetylase inhibitors, and HMG-CoA reductase inhibitors. Interestingly, many of these small molecules are oncology drugs. This result correlates with that last obtained with the network analysis because of the close relationship that may occur between the miRNAs involved in CVD and cancer. In this context, hesperidin has been established to have potential use in cancer therapy [132]. Furthermore, this is the first study proposing candidates for the treatment of CVD using miRNA-based and network pharmacology approaches, compared to previous studies using virtual screening and network pharmacology approaches [133]. This approach would open new possibilities for finding new candidates to treat CVD.

The employing of corticosteroids in cardiovascular diseases has been controversial because it has been associated with adverse cardiac effects such as cardiac hypertrophy, cardiac fibrosis, and cardiac remodeling [134]. However, Ospina-Quintero et al. [135] found that a low dose of vitamin D and dexamethasone can protect against heart disease when administered subcutaneously and repeatedly. This is because it induces the production of IL-10 by a network of lymphoid and myeloid immune cells. Also, Wang et al. [136] indicated that administering low doses of dexamethasone yielded cardioprotective effects in myocardial ischemia/reperfusion (MIR) mice. These effects were manifested through the enhancement of cardiac function, attenuation of reactive oxygen species (ROS), suppression of inflammatory responses, and stimulation of anti-oxidant responses. Additionally, the study by Wang et al. revealed that the protective mechanisms of dexamethasone on myocardial tissues were mediated through the Keap1/Nrf2/HO-1 pathway. [136]. Therefore, dexamethasone could have a protective effect on cardiovascular diseases, including CVD. However, more studies are necessary to support these hypotheses. These analyses may also be carried out at a clinical level, along with the other corticosteroids in this study.

Curcumin and hesperidin share many miRNAs. Hesperidin, a flavonone predominantly found in citrus fruits, is proposed to have the potential to serve as a therapeutic agent for the modulation of several cardiovascular diseases [58]. Curcumin demonstrates substantial efficacy in mitigating cardiomyocyte injury after ischemia and hypoxia, as well as in inhibiting myocardial hypertrophy and fibrosis, ameliorating ventricular remodeling, and attenuating drug-induced myocardial injury. Furthermore, it shows promise in improving diabetic cardiomyopathy (DCM), alleviating vascular endothelial dysfunction, impeding foam cell formation, and reducing the proliferation of vascular smooth muscle cells (VSMCs). Clinical studies have substantiated the protective impact of curcumin on blood vessels, while toxicological investigations have confirmed its safety profile. Nonetheless, it is imperative to acknowledge that elevated doses of curcumin may induce adverse effects, including hepatic impairment and perturbations in embryonic heart development [137]. These characteristics could propose curcumin as a drug with therapeutic potential for CVD.

Reversine, alternatively known as 2-(4-morpholinoanilino)-6-cyclohexylaminopurine, is a derivative of 2,6-disubstituted purine. This diminutive molecule exhibits encouraging antitumor characteristics by impeding diverse kinases that govern the cell cycle and cytokinesis. Through the application of network pharmacology prediction, molecular docking, and systematic review, it has been determined that MEK1 could potentially serve as a promising target for the action of reversine [138]. MEK1-ERK1/2 pathways are part of the MAPK cascade and have been implicated in regulating myocyte survival following ischemia–reperfusion injury, oxidative stress, and anthracycline exposure. These pathways have been associated with cardioprotective effects by directly antagonizing myocyte apoptosis [139] and play a crucial role in the signaling and transcriptional pathways that contribute to the pathogenesis of heart and vascular disease via the MEK-ERK-Egr-1 axis [140]. These data open a new line of study on miRNAs as potential biomarkers of CVD, genes, and contaminants or drugs that could be involved in developing or treating CVD.

## 5. Conclusions

Despite efforts to compile the available information on miRNAs and CVD, this review has limitations. Among the challenges encountered were the following: the studies were heterogeneous and there was an absence of pertinent clinical data, as well as a lack of a comprehensive survey on individual lifestyles, ethnicities, sample collection, methodologies, heterogeneity in protocols, miRNA isolation, comorbidities, and servers used for miRNA analysis. Additional epidemiological research is urgently required to comprehend the potential of miRNAs as a treatment for CVD or as a prospective risk factor for its development. Furthermore, a notable absence of Latin American representation in cardiovascular disease (CVD) profiles has been observed, underscoring the necessity of incorporating this demographic in forthcoming research endeavors. Notwithstanding these constraints, our study meticulously consolidated the foremost findings, delineating miRNA profiles prevalent in CVD. Our in silico strategies suggest possible associations between CVD and xenobiotic agents, including 5-Aza-CdR, arsenic, trichostatin A, and endogenous hormones.

Furthermore, the network analysis allowed us to identify potential drug-like compounds that share targeted miRNAs by hesperidin: desoximetasone, curcumin, flurandrenolide, fludrocortisone, diflorasone, and reversine. These compounds may be investigated in future preclinical or clinical studies to treat CVD. It is crucial to investigate these drugs deeper. Furthermore, the clinical use of miRNAs as biomarkers for the diagnosis and prognosis of CVD should be supported.

## Figures and Tables

**Table 1:** The pathways identified based on enrichment analysis of the dataset of genes involved in CVD.

Pathway or Process	XD Score	q-Value	Overlap/Size
Activation of the AP1 family of transcription factors	0.38079	1	1/10
Activation of chaperones by IRE1 alpha	0.38079	1	1/10
P38MAPK events	0.31412	1	1/12
Amino acid transport across the plasma membrane	0.29702	1	1/13
Activated TAK1 mediation of p38 MAPK activation	0.29702	1	1/13
Cytochrome p450 arranged by substrate type	0.28848	1	1/13
Pyrimidine metabolism	0.28848	1	1/13
Intrinsic pathway for apoptosis	0.2665	1	2/28
Activation of BH3-only proteins	0.24745	1	1/15

**Table 2:** Main features of the small molecules: curcumin and hesperidin.

Small Molecule	Pharmacological Activity	Shared miRNAs	Functions
Curcumin CID 969516 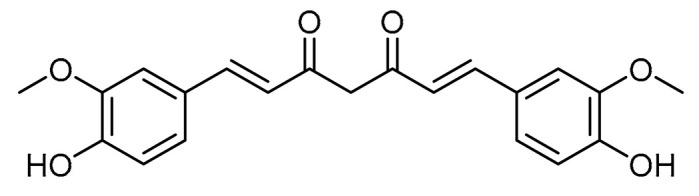	Polyphenol has multiple signaling targets and activity at the cellular level, and it has multiple health benefits [49]. It positively affects inflammatory conditions, metabolic syndrome, and pain, and helps manage inflammatory and degenerative eye conditions and kidneys [50].	miR-1	It plays a crucial role in the physiological processes of smooth and skeletal muscles and other tissues, thereby contributing to the pathogenesis of a broad spectrum of disorders [51].
		miR-29b	This miRNA regulates PTEN gene expression by downregulating DNMT expression and hypomethylating the PTEN promoter, resulting in tumor-suppressive effects [52].
		miR-324-5p	This miRNA is implicated in cell growth, apoptosis, cancer, epilepsy, neuronal differentiation, psychiatric conditions, cardiac disease pathology, and other physiological processes [53,54,55,56,57].
Hesperidin CID 10621 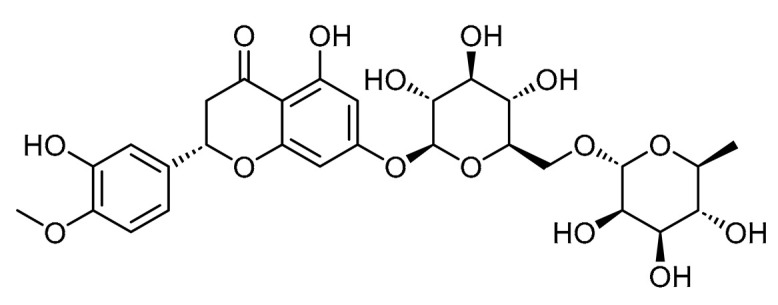	This compound is used to treat several blood vessel disorders, such as hemorrhoids, varicose veins, and impaired circulation (venous stasis). It also has demonstrated anticancer and hepatoprotective activities [58].	miR-374	It involves malignant processes like digestive system carcinoma, including proliferation, apoptosis, invasion, and metastasis [59].
		miR-197	It plays a crucial role in the post-transcriptional regulation of gene expression in multicellular organisms by influencing both the stability and translation of mRNAs [60].
		miR-467b	This miRNA reduces hepatocyte lipid accumulation by directly binding to the 3′UTR of Gpat1 and inhibiting GPAT1 expression at the mRNA and protein levels [61].
		miR-291b-5p	It participates during the preimplantation stage of mouse embryo development; the inhibition of autophagy is achieved by targeting Atg5 and Becn1 [62].

**Table 3:** The most outstanding small molecules with CVD-related miRNAs from the pharmacology network analysis (the score is depicted as a node with more neighbors).

Name	Activity	Score
5-fluorouracil	This pyrimidine derivative serves as an antineoplastic agent. 5-FU demonstrates a broad-spectrum antimicrobial activity and effectively inhibits virulence factors and biofilm production [63].	260
Glucose	The 6-carbon structure is a prevalent energy source for all organisms globally and plays a pivotal role in facilitating aerobic and anaerobic cellular respiration [64].	83
Cisplatin	The platinum-based drug was initially approved as a cancer-fighting agent. It exerts its antitumor effects through various cytotoxic mechanisms, including DNA damage and dysfunction of cytoplasmic organelles, particularly the endoplasmic reticulum and mitochondria [65].	59
Ginsenoside Rh2	A compound extracted from ginseng is monomeric and possesses anticancer activities both in vitro and in vivo [66].	52
Formaldehyde	Xenobiotic air pollutants and their universal distribution lead to widespread human exposure [67].	43
Diethylstilbestrol	It is a synthetic estrogen compound with an estimated potency approximately five times greater than endogenous estradiol. It shares structural analogies with other xenoestrogens and functions as an estrogen receptor-alpha (ERa) agonist with an affinity comparable to estradiol [68].	41
Enoxacin	Second-generation synthetic quinolone is recognized for its antibacterial activity. However, recent studies have focused on its potential as an anticancer agent [69].	35
Atorvastatin	A drug commonly used to lower cholesterol by inhibiting HMG-CoA reductase [70].	34
Arsenic trioxide	First-line treatment for acute promyelocytic leukemia is also effective for other types of leukemia [71].	32
Vorinostat (SAHA)	This histone deacetylase inhibitor treats advanced and refractory cutaneous T-cell lymphoma [72].	30
17beta-estradiol (E2)	The principal circulating estrogen hormone plays a crucial role in diverse physiological functions, encompassing the development and maintenance of numerous tissues and organs [73].	29
Hydroxycamptothecin (HCPT)	An indole alkaloid derived from the Chinese tree *Camptotheca acuminate* has been found to inhibit the activity of topoisomerase I effectively. This compound demonstrates a wide-ranging spectrum of anticancer activity in vitro and in vivo [74].	29
Doxorubicin	It is a chemotherapeutic agent employed in treating a diverse array of cancers. Its mechanism of action involves targeting multiple intracellular processes: generation of reactive oxygen species, formation of DNA adducts, inhibition of topoisomerase II, eviction of histones, regulation of Ca2+ and iron hemostasis, and overproduction of ceramide [75].	29
Emodin	It is primarily used in traditional Chinese medicine to treat carbuncles, sore throats, blood stasis, sores, and damp-heat jaundice. In recent years, pharmacological research has uncovered other potential therapeutic uses, including anticancer, neuroprotective, antidiabetic, anti-oxidant, and anti-inflammatory properties [76].	27
Bicalutamide	It is a potent non-steroidal androgen receptor blocker known for its peripheral selectivity, attributed to poor blood–brain barrier penetration. While its primary usage is in cisgender men with prostate cancer, it has also been employed in the treatment of rare cases of peripheral precocious puberty in boys [77].	23
Trichostatin A (TSA)	It is a natural derivative of dienohydroxamic acid derived from a fungal metabolite and exhibits various biological activities. It exerts antidiabetic activity and reverses high glucose levels by downregulating brain-derived neurotrophic factor (BDNF) expression in Schwann cells. [78].	23
Ascorbate	It is a potent anti-oxidant and a water-soluble vitamin [79].	21
Glucocorticoid	It is a steroid hormone commonly employed in treating autoimmune disorders, inflammation, and cancer [80].	20
All-trans-retinoic acid (ATRA)	It is an effective agent for preventing cancer and treating skin diseases and acute promyelocytic leukemia (APL). Its pharmacological effects are mainly mediated by retinoid X receptors (RXRs) and retinoic acid receptors (RARs) [81].	18
Mistletoe lectin-I	This lectin is the most crucial galactoside-specific lectin. Comprising a cytotoxic A-chain (29 kDa) and a distinct carbohydrate-binding B-chain (34 kDa), it exhibits immunomodulatory functions [82].	14
Sorafenib	It represents the first-line therapy for advanced hepatocellular carcinoma. Esteemed for its robust efficacy, this drug enhances overall survival rates [83].	14
Lenalidomide	It has been used as the standard therapy for multiple myeloma and other hematological diseases [84].	13
Ethanol	It is a natural byproduct of fermentation and a crucial chemical in the industrial sector. Recently, it has gained recognition as a valuable biofuel [85].	13
Docetaxel	It is a cytotoxic agent employed in the treatment of patients with breast cancer [86].	13
Gemcitabine	It is an anticancer agent used to treat a wide range of challenging tumors, such as breast, lung, pancreatic, and ovarian cancers [87].	12
Trastuzumab	This compound is used as first-line therapy to treat metastatic breast cancer related to HER2 overexpression [88].	11
Catechin	This agent affects the molecular mechanisms of cancer and related disorders, including angiogenesis, extracellular matrix degradation, cell death regulation, and multidrug resistance. Many studies have shown that drinking green tea improves heart health because of its several helpful effects, including anti-inflammatory, anti-oxidant, and antithrombogenic properties [89].	10
1alpha-25-Dihydroxyvitamin D3	Calcitriol, which has been identified as an active form of vitamin D3, serves as an activator of PKC and VDR [90].	9
Progesterone	This hormone is an endogenous steroid derived from cholesterol via pregnenolone. It is a primary gonadal hormone, predominantly synthesized in the corpus luteum of the ovaries and the placenta during pregnancy [91].	9

**Table 4:** The most outstanding candidate small molecules that share miRNAs targeted by hesperidin in CVD from the pharmacology network analysis (QED index shows that oral route can be used to administer a drug candidate).

Name	CID	QED Index	Degree	Betweenness	Molecule Class
Desoximetasone	5311067	0.787	3	0.000821	Corticosteroid
Curcumin	969516	0.698	1	0	Diarylheptanoid
Flurandrenolide	15209	0.697	4	0.002794779	Corticosteroid
Trifluridine	6256	0.695	4	0.003463385	Antiviral
Fludrocortisone	31378	0.678	5	0.002754087	Corticosteroid
Diflorasone	71415	0.658	6	0.003861498	Corticosteroid
Reversine	210332	0.513	15	0.025602386	Purine derivative
Tamoxifen	2733526	0.471	1	0	Estrogen receptor modulator
Vorinostat	5311	0.387	3	8.21 × 10^4^	Histone deacetylase inhibitor
(3R,5S)-Atorvastatin	46780495	0.212	12	0.013398551	HMG-CoA reductase inhibitor
Atorvastatin, (3S,5R)-	6093359	0.212	10	0.007799482	HMG-CoA reductase inhibitor
ent-Atorvastatin	62976	0.212	7	0.002740719	HMG-CoA reductase inhibitor
Atorvastatin	60823	0.212	12	0.013398551	HMG-CoA reductase inhibitor

## Data Availability

The data presented in this study are available in this article and Supplementary Materials.

## Supplementary Materials

The following supporting information can be downloaded at https://www.mdpi.com/article/10.3390/jox14040083/s1, Figure S1: Network analysis of CVD-associated miRNAs with their expression, sources, countries of origin, and detection methods; Figure S2: Network analysis of miRNAs associated with CVD and their predicted targets; Figure S3: The structural network represents the ten most connected nodes, including miRNAs and targets. Figure S4: The structural network of small molecules, phlebotonic, and miRNAs. Figure S5: Structural network of hesperidin-specific miRNAs that are also altered by small molecules. Table S1: CVD bibliography dataset; Table S2: Sources, countries, and methods; Table S3: miRNAs, targeted genes and their regulation; Table S4: Most connected nodes of genes and miRNAs; Table S5: Enrichment analysis of the most outstanding genes; Table S6: miRNAs and targeted small molecules; Table S7: Analysis of the most connected small molecules; Table S8: Results of network analysis of miRNAs and small molecules; Table S9: The most connected nodes between hesperidin, small molecules and miRNAs; Table S10: Druglikeness determined by QED index of the small molecules that shared the miRNA expression profile with hesperidin.
