# Supplementary material for: Network Pharmacology Approaches Used to Identify Therapeutic Molecules for Chronic Venous Disease Based on Potential miRNA Biomarkers
Source: J Xenobiot. 2024 Oct 15;14(4):1519–40. doi: 10.3390/jox14040083 (PMC11503387; doi:10.3390/jox14040083)
Supplement: Supplementary file 1 [file jox-14-00083-s001.zip › Supplementary Figure S1.pdf]

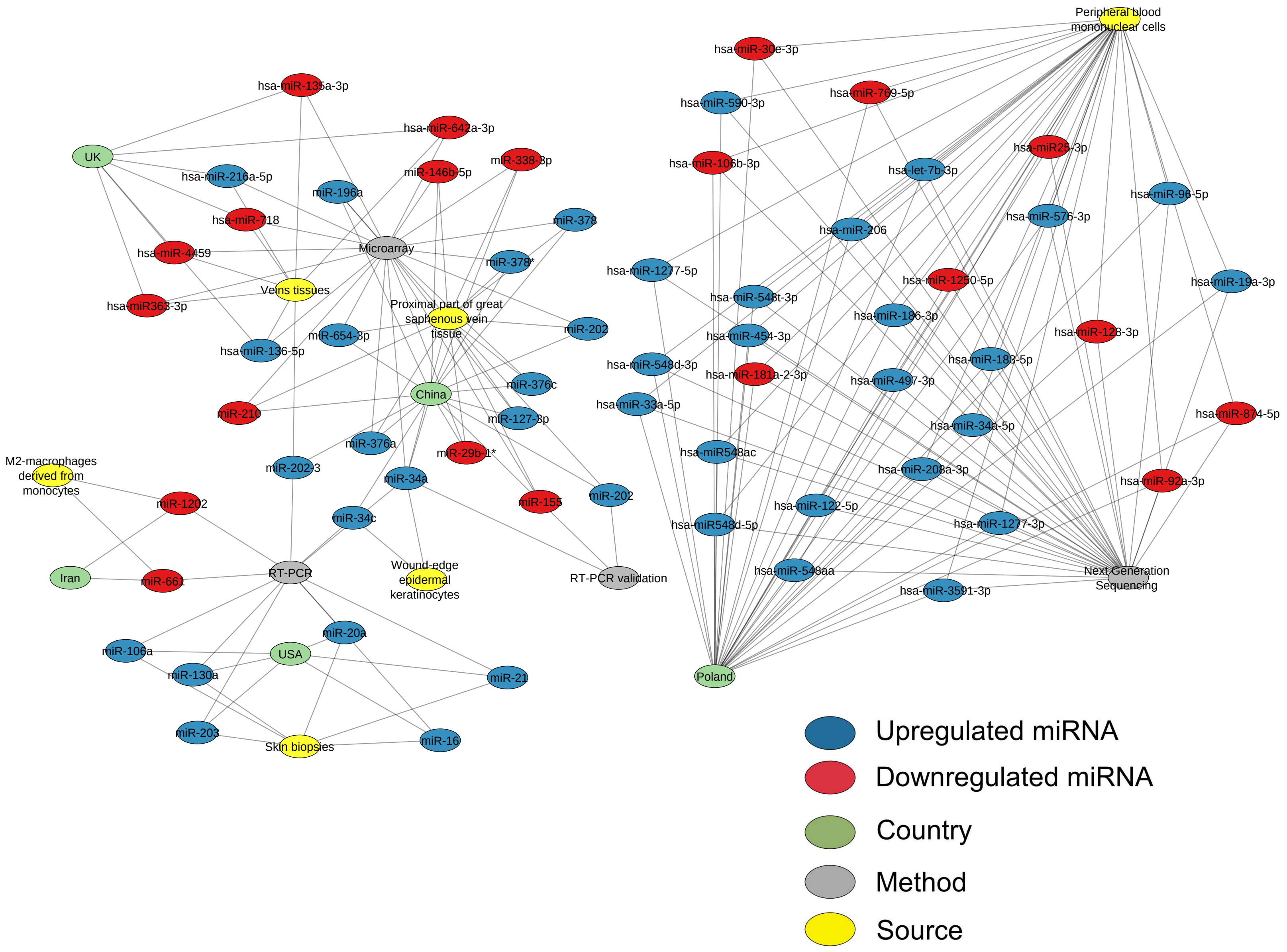

Figure S1. Network analysis of CVD-associated miRNAs with their expression, sources, countries of origin, and detection methods. The network depicts the interconnected structure of miRNAs derived from CVD patients, organized by their expression, sources, countries of origin, and detection methods. In the network, miRNAs are denoted as blue (upregulated) or red (downregulated) nodes, green nodes represent countries, yellow nodes represent sources, and gray nodes represent de-tection methods. The connections between the nodes signify the frequency of independent study reports. The three most outstanding sources were the proximal part of the signifi-cant saphenous vein tissue, vein tissues, and peripheral blood mononuclear cells. China was the country with the most available finds from miRNAs. Microarrays and RT-PCR are the most effective methods for diagnosing CVD. At least two tissues are expected to contain four specific miRNAs: miR-34a, miR-34c, miR-202-3, miR-1202, and miR-130a. The network, constructed using Cytoscape software (v.3.10.2), comprises 78 nodes and 193 edges, with a diameter and a network density of 6 and 0.106, respectively.
