# Supplementary material for: Network Pharmacology Approaches Used to Identify Therapeutic Molecules for Chronic Venous Disease Based on Potential miRNA Biomarkers
Source: J Xenobiot. 2024 Oct 15;14(4):1519–40. doi: 10.3390/jox14040083 (PMC11503387; doi:10.3390/jox14040083)
Supplement: Supplementary file 1 [file jox-14-00083-s001.zip › Supplementary Figure S2.pdf]

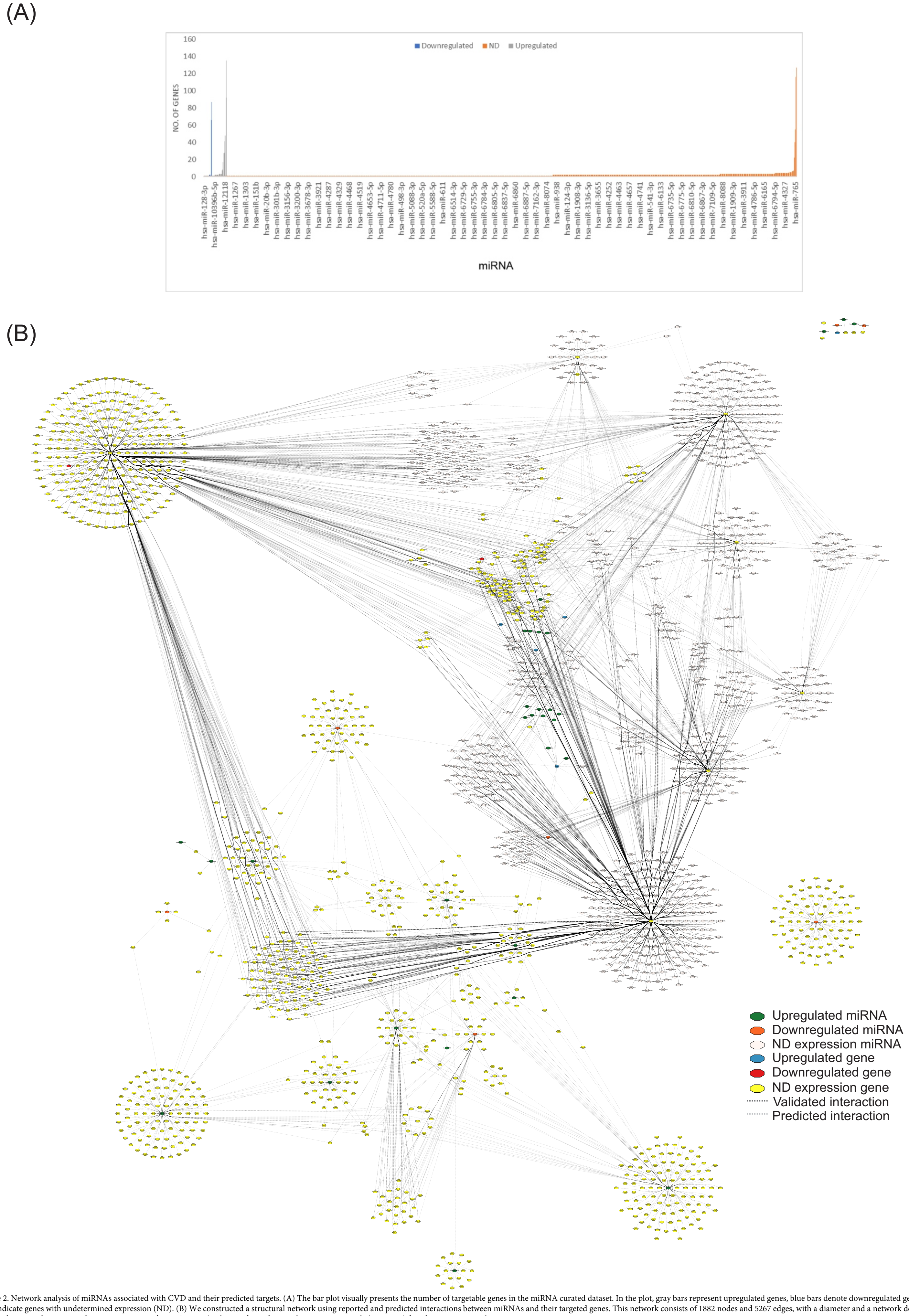


Figure 2. Network analysis of miRNAs associated with CVD and their predicted targets. (A) The bar plot visually presents the number of targetable genes in the miRNA curated dataset. In the plot, gray bars represent upregulated genes, blue bars denote downregulated genes, and orange bars indicate genes with undetermined expression (ND). (B) We constructed a structural network using reported and predicted interactions between miRNAs and their targeted genes. This network consists of 1882 nodes and 5267 edges, with a diameter and a network density of 12 and 0.001. The network was created using Cytoscape software (v.3.10.2). Please refer to the Supplementary Materials (Figure S2) for a better image resolution.
