# Supplementary material for: Network Pharmacology Approaches Used to Identify Therapeutic Molecules for Chronic Venous Disease Based on Potential miRNA Biomarkers
Source: J Xenobiot. 2024 Oct 15;14(4):1519–40. doi: 10.3390/jox14040083 (PMC11503387; doi:10.3390/jox14040083)
Supplement: Supplementary file 1 [file jox-14-00083-s001.zip › Supplementary Figure S3.pdf]

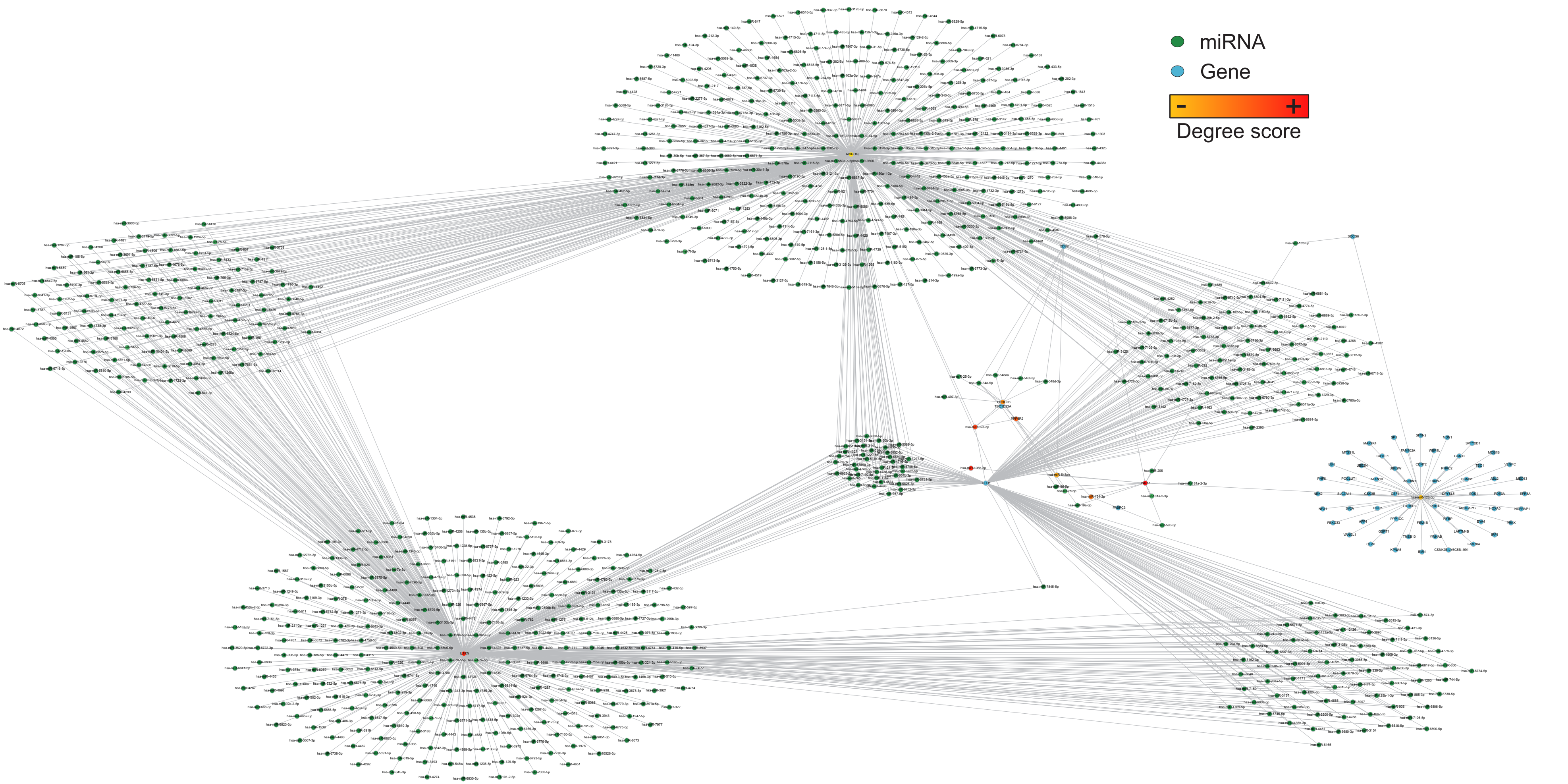

Figure S3. The structural network represents the ten most connected nodes, including miRNAs and targets. Nodes are color-coded from orange to yellow based on their degree of connection, representing the most connected genes and miRNAs in the network. The most relevant nodes in this network are WNK-1, hsa-miR-106b-3p, IL1NR, hsa-miR-92a-3p, PPP6R2, hsa-miR-454-3p, PRRC2B, hsa-miR-548ac, hsa-miR-128-3p, and ADIPOQ. The network consists of 921 nodes and 1256 edges, with a diameter and a network density of 7 and 0.003, respectively. This network was created using Cytoscape software (v.3.10.2).
