# Supplementary material for: Network Pharmacology Approaches Used to Identify Therapeutic Molecules for Chronic Venous Disease Based on Potential miRNA Biomarkers
Source: J Xenobiot. 2024 Oct 15;14(4):1519–40. doi: 10.3390/jox14040083 (PMC11503387; doi:10.3390/jox14040083)
Supplement: Supplementary file 1 [file jox-14-00083-s001.zip › Supplementary Figure S4.pdf]

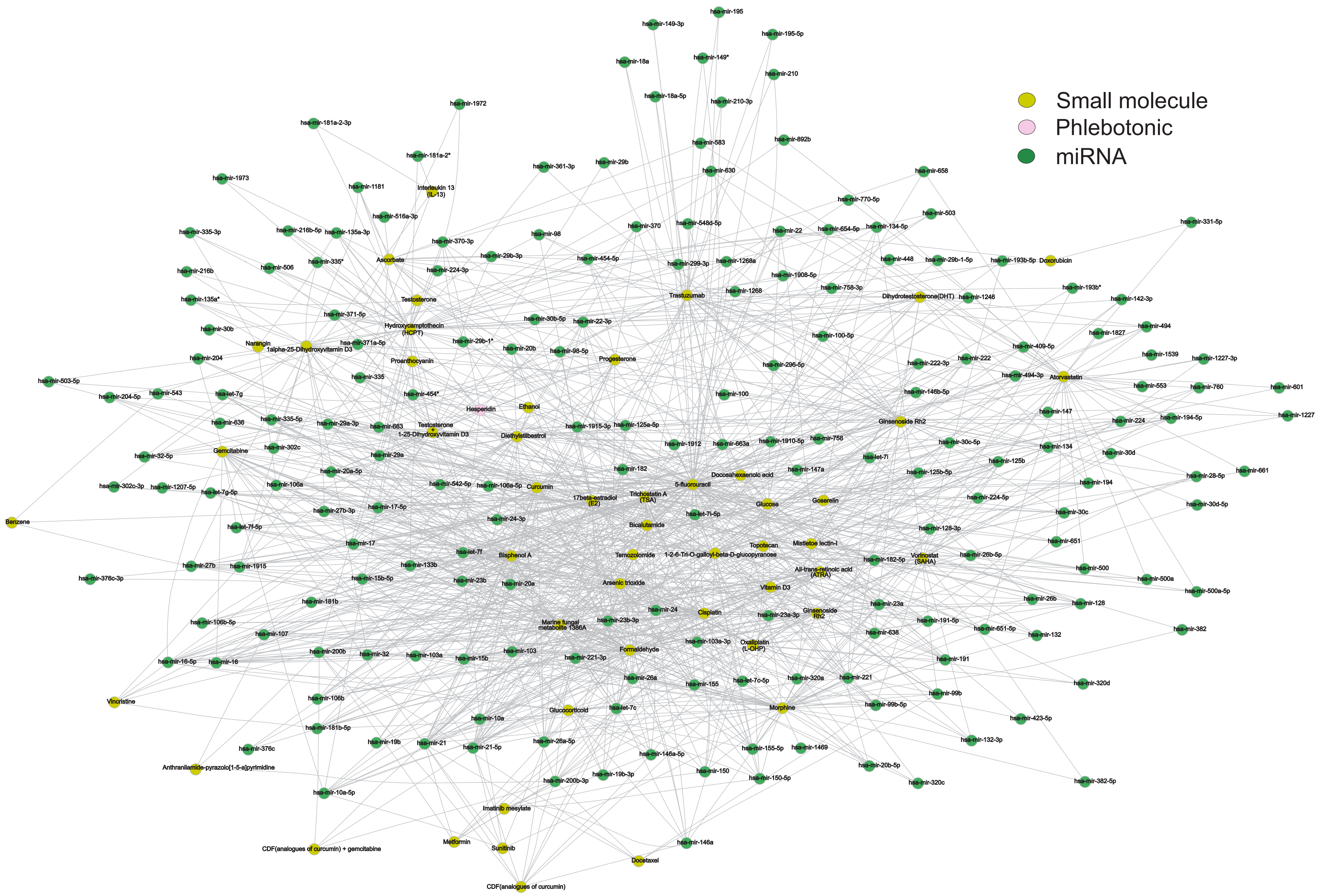


Figure S4. Figure 4. The structural network of small molecules, phlebotonic, and miRNAs. The structural network depicts the targeted miRNAs (green nodes) between small molecules (yellow nodes) and the phlebotonic hesperidin (pink node). It was observed that curcumin exhibited the highest connection of miRNAs with the reported phlebotonic hesperidin. The network involves 246 nodes and 1153 edges, with a diameter and a net-work density of 6 and 0.038, respectively. The network construction utilized Cytoscape software (v.3.10.2).
