# Supplementary material for: Network Pharmacology Approaches Used to Identify Therapeutic Molecules for Chronic Venous Disease Based on Potential miRNA Biomarkers
Source: J Xenobiot. 2024 Oct 15;14(4):1519–40. doi: 10.3390/jox14040083 (PMC11503387; doi:10.3390/jox14040083)
Supplement: Supplementary file 1 [file jox-14-00083-s001.zip › Supplementary Figure S5.pdf]

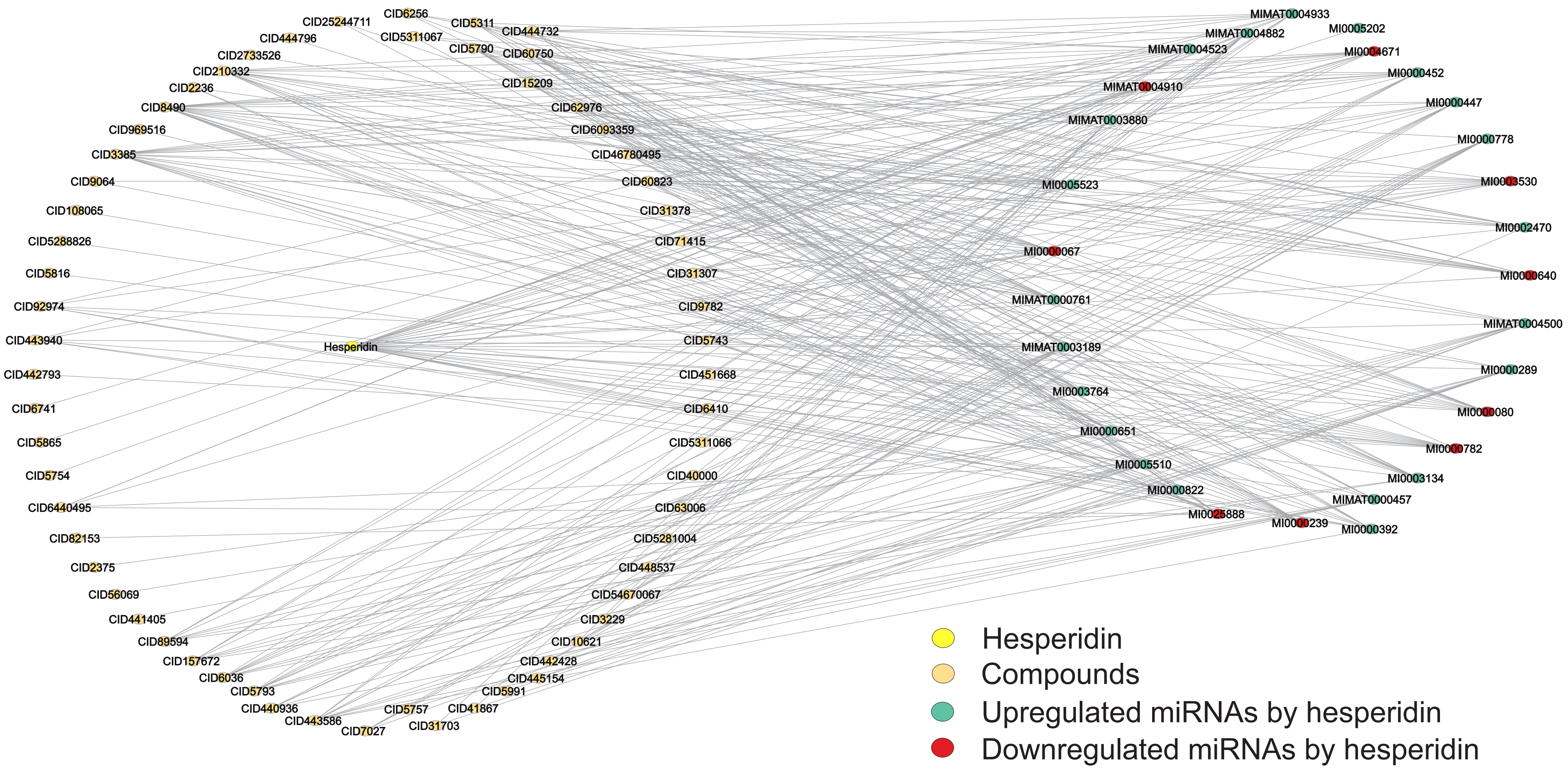

Figure S5. Structural network of hesperidin-specific miRNAs that are also altered by small molecules. This structural network depicts the miRNA profiles shared by both miRNAs that are specifically upregulated or downregulated by the reference compound hesperidin and the small molecules studied in this work. These shared miRNA profiles between hesperidin and small molecules allow the selection of potential candidates for CVD treatment. Downregulated miRNAs are shown in red, upregulated in blue, and those shared with small molecules in orange. The network involves 93 nodes and 320 edges, with a diameter and a density of 4 and 0.075. respectively.
